# Characterization of the complete chloroplast genome of *Camellia anlungensis*

**DOI:** 10.1080/23802359.2020.1716639

**Published:** 2020-02-04

**Authors:** Yayan Zhu, Jie Xu, Gang Wang, Congjun Yuan, Yang Luo, Xiaoyong Dai

**Affiliations:** Guizhou Academy of Forestry, Guiyang, Guizhou Province, People’s Republic of China

**Keywords:** *Camellia anlungensis*, high-throughput sequencing, chloroplast genome

## Abstract

*Camellia anlungensis* is a rare and ornamental plant. To contribute to its conservation, the complete chloroplast (cp.) genome of *C. anlungensis* was sequenced and assembled by high-throughput sequencing technology, and its characteristics were analyzed and clustering relationship was studied in the present study. The complete cp. genome of *C. anlungensis* is 156,587 bp in length, containing a LSC region of 86,262 bp, a SSC region of 18,339 bp and two IR regions of 25,993 bp. The overall A + T content of *C. anlungensis*cp genome is 62.7%. The annotated complete cp. genome contains 114 genes, including 80 protein-coding genes, 30 tRNA genes, and 4 rRNA genes. Phylogenetic analysis suggested that *C. anlungensis*is grouped with *C*. *leptophylla, C. ptilophylla*, *C. pubicosta*, *C. grandibracteata* and *C. sinensis*.

*Camellia anlungensis* belongs to the *Tuberculata* of *Calmellia*, The *Tuberculata*is named for its ovary and fruit surface with tuberculate protrusions and considered to be a special group with original characters in *Camellia* (Ming and Zhong 1993; Jiang 2009). Guizhou province is the distribution center of *Tuberculata* (Zhang et al. 1998), and *C. anlungensis*is endemic in Guizhou (Zhou et al. 2001; An 2005). *Camellia anlungensis* grow under or in gullies of evergreen broad-leaved forests at an altitude of 720–850 m, and mainly distribute in Ceheng, Wangmo, and Anlong where is the model production area(An 2005). *Camellia anlungensis* is a kind of ornamental plant with rarely big white flowers (Liu and Zhang 1998).

Compared with nuclear genome, cp. genome is small, stable in structure, highly conserved in sequence and low in genetic recombination rate, which is an important marker for the study of phylogenetic evolution of plants (Luo et al. 2018). The *C. anlungensis* specimen was collected from Guiyang (Guizhou, China) and the biopsy specimen was conserved at the Guizhou Academy of Forestry. To study the relationship of *C. anlungensis*, we assembled its cp. genome from Illumina sequencing reads, and the annotated genome sequence have been deposited into the GenBank under the accession number MN756594.

Cp. DNA was extracted and purified by the Biomarker Biotechnology Co. Led (Beijing, China). The high-throughput sequencing was carried out by the Illumina HiSeq X Ten. Raw data were filtered and cp. reads were extracted from clean data to assembly by MITObimv1.9 (Hahn et al. 2013). Genome mapping was performed using the OGDraw online tool (Lohse et al. 2013) and phylogenetic trees basing on maximum likelihood method was constructed by the MEGA7 software (Kumar et al. 2016).

*Camellia anlungensis* cp. genome is a typical four-part structure with 156,587 bp in length. The content of GC is 37.3%, and the annotated complete cp. genome contains 114 genes, including 80 protein-coding gene (PCG), 30 transfer RNA (tRNA), and 4 ribosomal RNA(rRNA). Among all the genes, 19 genes occur in double copies, containing 8 PCG (ndhB, rpl2, rpl23, rps7, rps12, ycf1, ycf2 and ycf15), 7 tRNA genes (trnA-UGC, trnH-CAU, trnI-GAU, trnL-CAA, trnN-GUU, trnR-ACG andtrnV-GAC), and 4 rRNA genes (rrn4.5, rrn5, rrn16 and rrn23),and 18 genes contains introns, 16 genes contain only one intron (10 protein-coding genes and 6 tRNA genes), and the genes of clpP and ycf3contain 2 introns.

Because of the controversy over the interspecific relationship of the genus *Camellia*, the phylogenetic relationship among the 30 *Camellia* species were investigated by a maximum-likelihood (ML) phylogeny to construct a neighbor-Joining tree (Figure 1). The results showed that *C. anlungensis* is phylogenetically related to *C. leptophylla*, *C. ptilophylla, C. pubicosta, C. grandibracteata* and *C. sinensis*.

**Figure 1.**
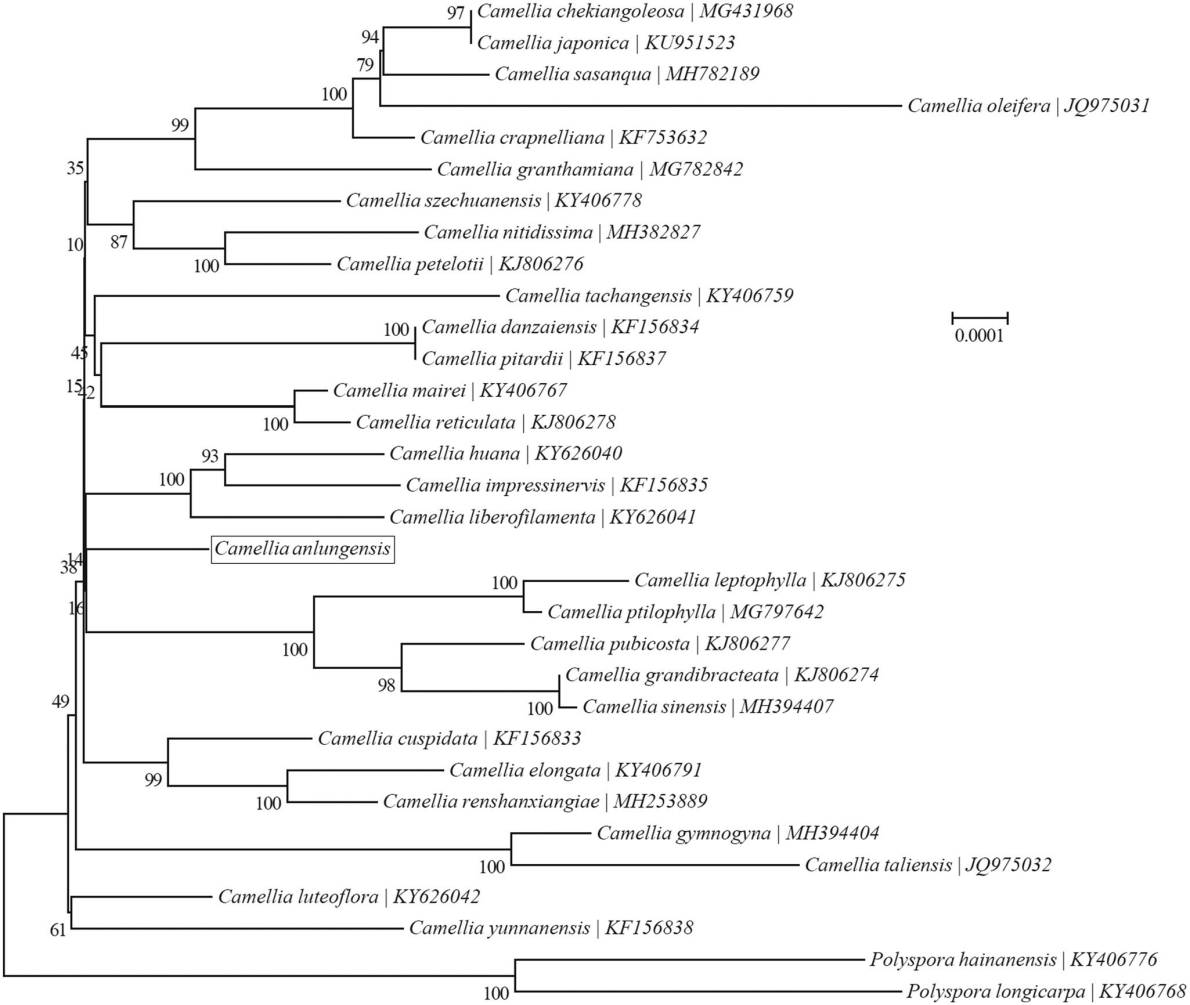
Phylogenetic relationships of 32 species based on the maximum-likelihood (ML) analysis of cp. PCGs.
